# The Influence of Tool Shape and Process Parameters on the Mechanical Properties of AW-3004 Aluminium Alloy Friction Stir Welded Joints

**DOI:** 10.3390/ma14123244

**Published:** 2021-06-11

**Authors:** Anna Janeczek, Jacek Tomków, Dariusz Fydrych

**Affiliations:** Institute of Machines and Materials Technology, Faculty of Mechanical Engineering and Ship Technology, Gdańsk University of Technology, Gabriela Narutowicza Street 11/12, 80-233 Gdańsk, Poland; anna.janeczek@pg.edu.pl (A.J.); dariusz.fydrych@pg.edu.pl (D.F.)

**Keywords:** FSW, aluminium alloy, visual testing, tensile strength test

## Abstract

The purpose of the following study was to compare the effect of the shape of a tool on the joint and to obtain the values of Friction Stir Welding (FSW) parameters that provide the best possible joint quality. The material used was an aluminium alloy, EN AW-3004 (AlMn1Mg1). To the authors’ best knowledge, no investigations of this alloy during FSW have been presented earlier. Five butt joints were made with a self-developed, cylindrical, and tapered threaded tool with a rotational speed of 475 rpm. In order to compare the welding parameters, two more joints with a rotational speed of 475 rpm and seven joints with a welding speed of 300 mm/min with the use of a cylindrical threaded pin were performed. This involved a visual inspection as well as a tensile strength test of the welded joints. It was observed that the value of the material outflow for the joints made with the cylindrical threaded pin was higher than it was for the joints made with the tapered threaded pin. However, welding defects in the form of voids appeared in the joints made with the tapered threaded tool. The use of the cylindrical tool resulted in higher values for about 37% of mechanical properties compared with the highest result for the tapered threaded joint. As far as the parameters were concerned, it was concluded that most of the specimens were properly joined for a rotational speed of 475 rpm. In the joints made with a welding speed of 300 mm/min, the material was not stirred properly. The best joint quality was given for a rotational speed of 475 rpm as well as a variety of welding speed values between 150 and 475 mm/min.

## 1. Introduction

Aluminium alloys are widely applied in industries such as automotive, aerospace, offshore and shipbuilding due to their relatively superior mechanical properties [1]. Their greatest advantages are very high corrosion resistance, high fatigue strength, low cost, but most of all, low density, all of which suit the requirements of the above-mentioned industries [2,3,4]. The aluminium alloys are commonly joined using inert gas arc welding processes and laser beam welding processes [5,6,7]. However, conventional methods are limited as aluminium alloys form a film of high-melting aluminium oxide on their surface very easily, and this needs to be removed before welding. Moreover, it is difficult to heat the material locally due to its high heat conductivity and coefficient thermal expansion values, which also makes the joint prone to stresses and deformation [8,9]. This is why the solid-state welding method is recommended with a view to obtaining very high mechanical joint properties.

The Friction Stir Welding (FSW) method is becoming more popular due to the very high quality of the joints being produced. It is even comparable to laser beam welding [10,11]. Moreover, the FSW does not require any filler metal, which makes the joint lighter [12,13]. It is also a significant factor, both in attaining the optimum vehicle speed and controlling fuel consumption. Furthermore, in comparison with the conventional arc welding methods, the FSW process has been proven to consume less power and has a lower maximum load [14]. The main important parameter associated with the method is the temperature of the welded material. The purpose of the method is to carry it out on the material, reaching the temperature below its melting point. With this in mind, the process parameters, including rotational and traverse speed, cannot be too high, as the metal would melt. If the properties are too low, the metal is not stirred properly [15]. Heat is generated from the friction caused by the tool rotating between the joining materials. Therefore, the higher the rotational speed, the more heat is generated. The traverse speed spreads the heat of the material along the joint [16].

Zlatanovic et al. [17] compared the effect of different rotational speeds on joints made with Friction Stir Spot Welding. It was stated that for the AA5754-H111 aluminium alloy, higher mechanical properties were obtained at lower rotational speeds (1000–1500 rpm). Rotational speed is one of the parameters that have the highest impact on the tensile strength results [18]. The research also confirms the correlation between the rotational speed and the temperature of the welded joint. Verma and Misra [19] noted an increase in temperature of about 100 °C while the rotational speed was increased from 1325 to 1812 rpm. Furthermore, an increase in welding speed led to a decrease in temperature.

Apart from rotational and welding speeds, another significant parameter influencing the process is the shape of the tool used [20,21,22,23,24,25]. The FSW tool consists of a shoulder and pin, and both parts may come in a great variety of shapes [20]. Tamadon et al. [21] researched the influence of the shape of the tool pin on the AA1100 aluminium alloy joint. In this research, conical, square and cylindrical threaded shapes were used. The study showed that the highest tensile strength results were attained using the cylindrical threaded tool due to a higher level of plasticity caused by more intense contact between the tool and the welded material. Another comparative study examined the use of pins in the following shapes: cylindrical, cylindrical cam, tapered, tapered cam and square. Defect-free Al–Cu joints were produced using the cylindrical cam and square pins. However, higher mechanical properties were attained using the square pin [26]. In another study, the different diameters of the tool pin (3 and 4 mm) were examined using different welding parameters. It was concluded that the 4 mm pin accounted for approximately 5% higher mechanical properties [27]. Kaushik and Dwivedi [22] examined the effect of tool shoulder diameter on heat generation. They found that the 25 mm shoulder diameter generated the highest amount of heat, which caused cracking. It was also possible to weld using a bobbin tool that enabled both sides of the joint to be welded simultaneously for full penetration. This variation is called BT-FSW [28,29]. 

Besides the tools and welding parameters, there are many other methods of improving FSW joint quality. One example is water cooling, which reduces and controls the temperature of the joints, especially for highly conductive materials (e.g., aluminium alloys) [29,30]. A study by Bocchi et al. [30] shows that water cooling increases the hardness rate in the heat affected zone (HAZ) and thermo-mechanically affected zone (TMAZ) but also decreases the elongation of the joints. On the other hand, some materials need the opposite treatment to cooling as the joint can only be produced at high temperature values. An example of this is the dissimilar joint of NiTi/Ti_6_Al_4_V that was made defect-free using back-heating during FSW process. Without preheating, cracks appeared in the joints used [31]. Another way to improve the FSW method is to use the ultrasonic-assisted FSW variant. It has been proven that ultrasonic vibrations can decrease welding forces, especially the traverse force. However, it does not cause a decrease in the mechanical properties of the joints used [32]. Said variant was also found to increase tensile strength results and reduce the risk of defects arising from low heat input for Ti/Al joints [33].

As FSW is a solid-state welding process, defects such as porosity or cold cracking do not occur. The most common defects in aluminium alloys are voids, kissing bonds, flash defects (material flow), lack of penetration or cracks [34,35,36,37]. These defects are mainly caused by incorrect parameter selection. For example, flash defects occur when the material is too soft as too much heat is generated by friction (due to high rotational speed) and therefore flows outside the joint [36,38]. This is also related to pin plunge depth. It may occur when the depth is too high. On the other hand, voids tend to result from low heat generation which makes it impossible to stir welded materials properly. This can be solved by increasing rotational speed [39,40].

Using this method, it is possible to join a wide range of materials and make dissimilar joints [41]. Apart from aluminium alloys, the studies proved that copper, titanium and magnesium alloys, as well as steel and polymers [42,43,44,45,46,47], could also be joined. Iwaszko and Kudła [48] successfully performed a friction stir processing (FSP) process on a Cu/SiC composite. Based on the observation of micro-structural changes, it emerged that FSW enables an improvement in surface properties. Turkan and Karakas [43] developed and compared two finite element models for AZ31B alloy FSW joints. The temperature and strain distributions were also compared with the experimental method in order to choose the most accurate model. In addition, research into improving dissimilar joints also continues. A group of researchers from India and Russia aimed to explain the fracture mechanisms of aluminium and titanium joints. It emerged that uneven titanium flakes were the main cause of cracks in dissimilar joints [44]. Derazkola et al. [45] are working on the joining of thermoplastic materials. In their latest study, a model for polycarbonate joints was made and compared with the experiment. It emerged that an increase in rotational speed would provide higher joint quality of the joints without causing any cracks, due to the lower risk of the material used sticking to the tool used.

Considering the above-mentioned facts, the purpose of this research was to obtain the best mechanical properties possible for joining EN AW-3004 (AlMn1Mg1) aluminium alloy by FSW process. To the authors’ best knowledge, no studies of FSW joints of this alloy have been presented before. The issue was investigated by optimizing the welding parameters and comparing the FSW tool shape and its effect on the joints. During investigations, joints with different welding parameters were performed. Each joint was subjected to non-destructive and destructive tests to determine their mechanical properties as tensile strength and elongation. Investigated aluminium alloy grade is often used in the food packaging, production, architecture and automotive industries due to its high strength, workability, very high corrosion resistance and thermal stability [49,50].

## 2. Materials and Methods

### 2.1. Materials Used

The aluminium alloy chosen for testing was EN AW-3004 (AlMn1Mg1). The dimensions of the EN AW-3004 single plates were 170 mm × 60 mm × 5 mm. The chemical composition and mechanical properties of the aluminium alloy are presented in Table 1.

### 2.2. Welding Process

**Figure 1:**
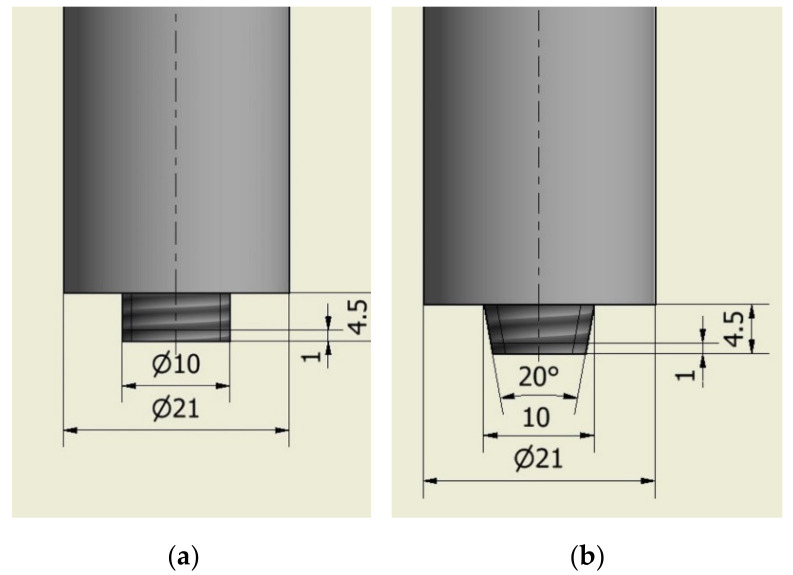
Sketch of tool pin shapes (dimensions in mm): (**a**) cylindrical threaded, (**b**) tapered threaded.

The welding process was performed on a milling machine (Metal Technics Polska s.c, Sokołów Podlaski, Poland) using a self-developed welding tool. Cylindrical threaded (Figure 1a) and tapered threaded (Figure 1b) shapes of the tool pins were used. The shapes are presented in Figure 1. In order to compare the effect of the shape of the welding tool used on the joint produced, five joints were made with each tool. To compare the welding parameters, nine more joints were made with the cylindrical threaded pin. A total of 19 butt joints were made in the flat welding position (PA). The tool plunge depth was 4.5 mm.

The parameters were selected on the basis of the high heat conductivity of the aluminium alloy plates [8,9] and high heat distribution obtained during FSW process [16]. Firstly, a rotational speed of 475 rpm was chosen as this was considered to be a low level that generated less heat [23]. The equal set of parameters were chosen to perform welding with different tool pin shapes. The same rotational speed (475 rpm) was maintained together with a variety of welding speeds ranging from 150 to 475 mm/min. On the basis of primary visual tests of the specimens made at 475 rpm, a welding speed of 300 mm/min was chosen for further parameter examinations. Seven joints were made using a cylindrical threaded pin at the above-mentioned welding speed as well as a variety of rotational speeds ranging from 115 to 925 rpm. Two additional joints were made at the same rotational speed (475 rpm) and welding speeds of 95 and 600 mm/min for more accurate results. The parameters used are presented in Table 2.

### 2.3. Examination Procedure

**Figure 2:**
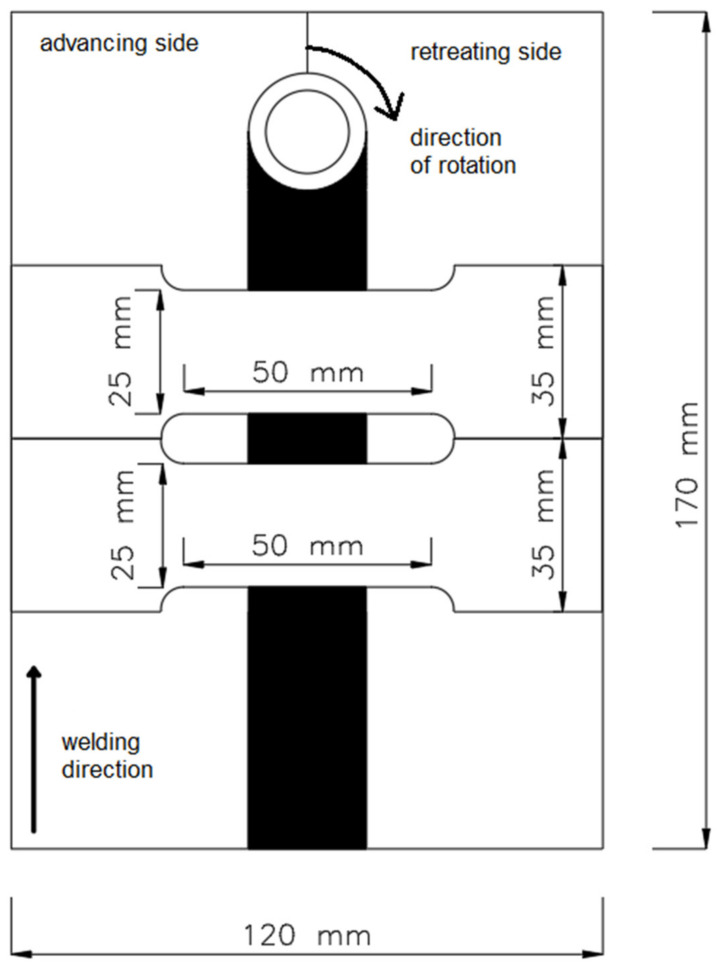
The location of the specimens cut for the tensile strength tests.

Specimens were investigated by non-destructive (NDT) and destructive tests (DT). Firstly, visual tests (VT) were performed in accordance with the EN ISO 17637:2017 standard [53]. The VT for the FSW joints revealed cracks, lack of penetration and overheating of the plates as well as a certain amount of material outflow [35,36,37]. This information was of particular importance as it made it possible to select appropriate welding parameters for producing joints. The next step was to cut the joints into specimens to be used for tensile strength tests. The location scheme for said specimens is presented in Figure 2. The tests were carried out in accordance with the EN ISO 6892-1:2020 standard [54].

From each joint, two specimens were cut crosswise to the longitudinal axis of the metal. Two joints (CW1 and CW7) were not tested for tensile strength as they broke while cutting. Before the tests could be carried out, the specimens needed to be cleaned of excessive material outflow. The tensile strength tests were performed using the ZD 100T (Jinan Hensgrand Instrument Co., Ltd, Jinan, China) tensile testing machine with a measurement range of up to 200 kN (20 T). The specimens were tested with a 2 T load.

On the basis of the tensile strength test results (maximum force measurements) obtained, the tensile strength (R_m_) and elongation (A_50_) values were calculated. The results were analyzed twice to compare the effect of the tool’s shape and welding parameters on the joint properties. Firstly, the analysis consisted of pointing out the highest and lowest values of the tensile strength test. This led to a comparison of the tool shapes. The next step was choosing the range of the parameters, which provide the highest tensile strength and biggest value of elongation.

## 3. Results and Discussion

### 3.1. Visual Tests (VT)

This section is divided into two parts: a comparison of the effects of different tool pin shapes on the joint produced and a comparison of the effects of different welding parameters on the joint produced. Every joint has defects in the form of craters at the beginning of the joint on the advancing side and in the exit hole at the end of the joint.

#### 3.1.1. Comparison of the Tool Pin’s Shape

**Figure 3:**
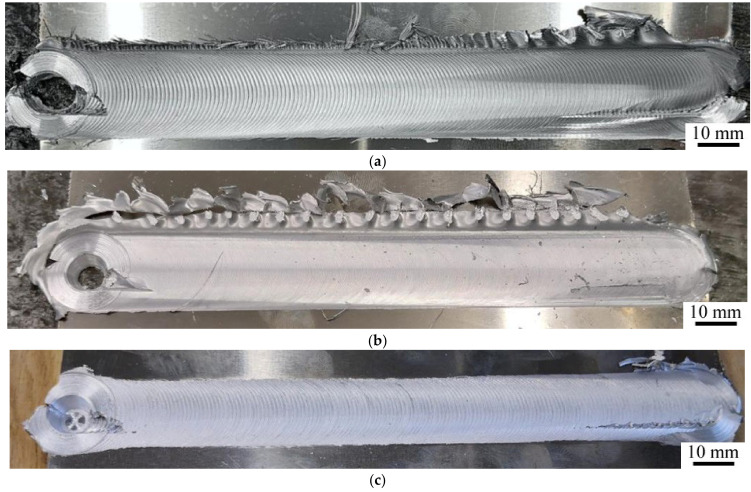
The FSW (Friction Stir Welding) joints: (**a**) CR6; (**b**) TR 1; (**c**) TR5.

Top views of exemplary joints are presented in Figure 3. During the tests, it was observed that pin shape had a significant impact on the appearance of the joint produced as more material outflow on the retreating side of the joint was seen in joints made with the cylindrical threaded pin. Material outflow is a common defect [24,35,55]. The amount of outflow is associated with the shape of the pin and the process parameters [17,21,24]. A comparison of the CR6 and TR5 joints made with equal parameters of 475 mm/min and 475 rpm revealed that the TR5 joint showed no material outflow while the CR6 showed a significantly greater amount of it, especially at the beginning of the joint (Figure 3a,c). This may have resulted from decreasing diameter of the tapered threaded pin that plasticized the material. As in that case, the amount of plasticized material was lower and was not emerging outside the weld comparing to the consistent diameter of cylindrical threaded pin. For joints made with the tapered threaded pin, only one joint showed any material outflow (TR1–Figure 3b), which suggest that used parameters were incorrect. The VT results prove that the tapered pin produces a joint with the lowest amount of material outflow.

#### 3.1.2. Comparison of the Welding Parameters

**Figure 4:**
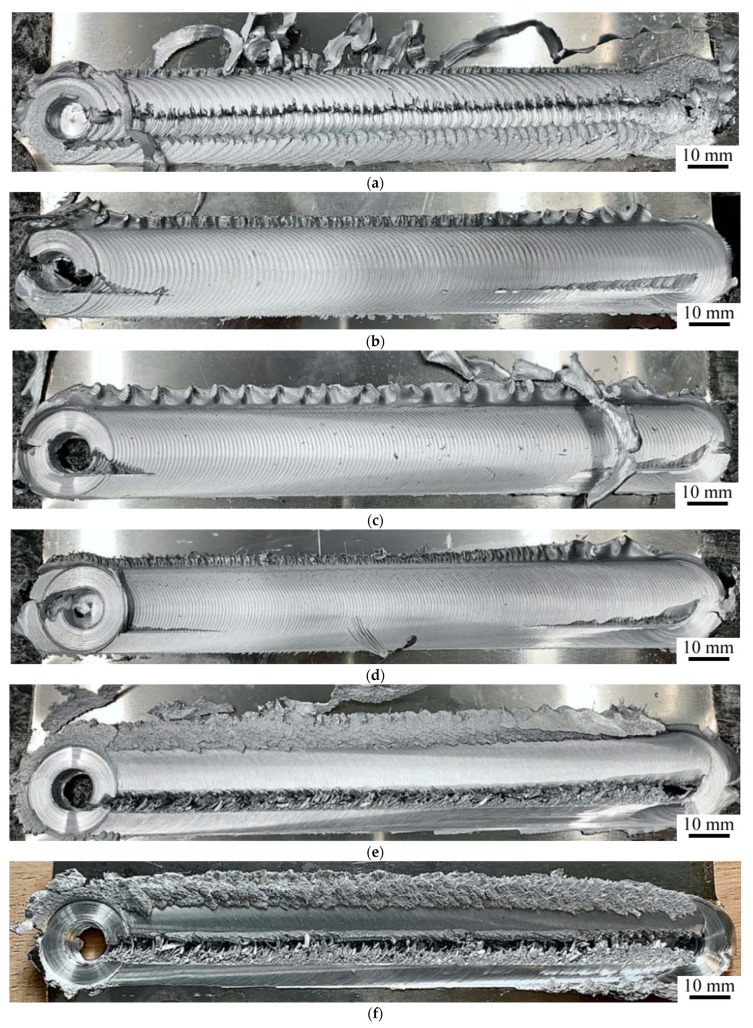

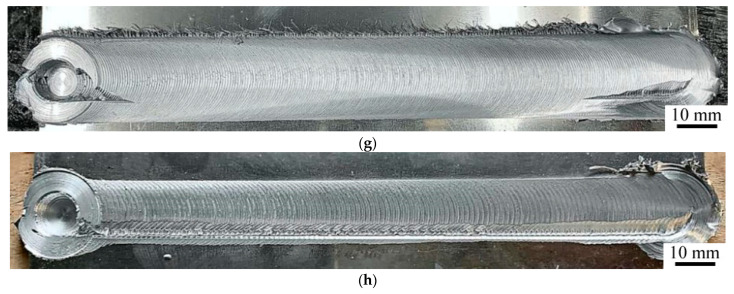
The FSW joints: (**a**) CW1; (**b**) CW3; (**c**) CW4; (**d**) CW5; (**e**) CW6; (**f**) CW7; (**g**) CR4; (**h**) CR7.

Views of sample joints are presented in Figure 4. The VT results suggest that welding parameters also have an impact on the presence of the joint. There are differences in the amount of material outflow, visibility of welding marks and the presence of a groove or lack of bonding. The lowest amount of material outflow was shown by the CR4 and CR7 specimens (Figure 4g,h). However, in CR7, a lack of bonding was revealed near the advancing side of the joint (Figure 4h). The same defect was also found in the CW1, CW6 and CW7 (Figure 4a,e,f). Those joints were made at rotational speeds of 115 (CW1), 680 (CW6) and 925 rpm (CW7). This suggests that the rotational speed was too low as the material was not sufficiently heated and stirred and confirms the findings of the previous research into the effect of insufficient rotational speed on heat input [56]. On the other hand, speeds of 680 and 925 rpm were too high for the welding speed of 300 mm/min as the material melted down. Moreover, in the CW3, CW4 and CW5 joints, cracks appeared at the end of the joint and in the exit hole (Figure 4b–d). The welding marks became more visible with the increase in welding speed or decrease in rotational speed.

### 3.2. Tensile Strength Tests

Like the previous section, this one also contains a comparison of tool pin shape and welding parameters. The tensile strength (Rm) and elongation (A_50_) results presented in this section amount to the average obtained for two specimens cut from a single joint, following the scheme in Figure 2. The average R_m_ result of the base material was 226 MPa and for A_50:_ 24%. During the tests, every specimen broke on the advancing side of the weld material, in the thermo-mechanically affected zone (TMAZ) or heat affected zone (HAZ). In the case of lap joints, the TMAZ-weld metal boundary (the stirring zone in the lap joint) was found to be the one where the initiation of fatigue cracking occurred due to plastic deformation [57].

#### 3.2.1. Comparison of the Tool Pin’s Shape

**Figure 5:**
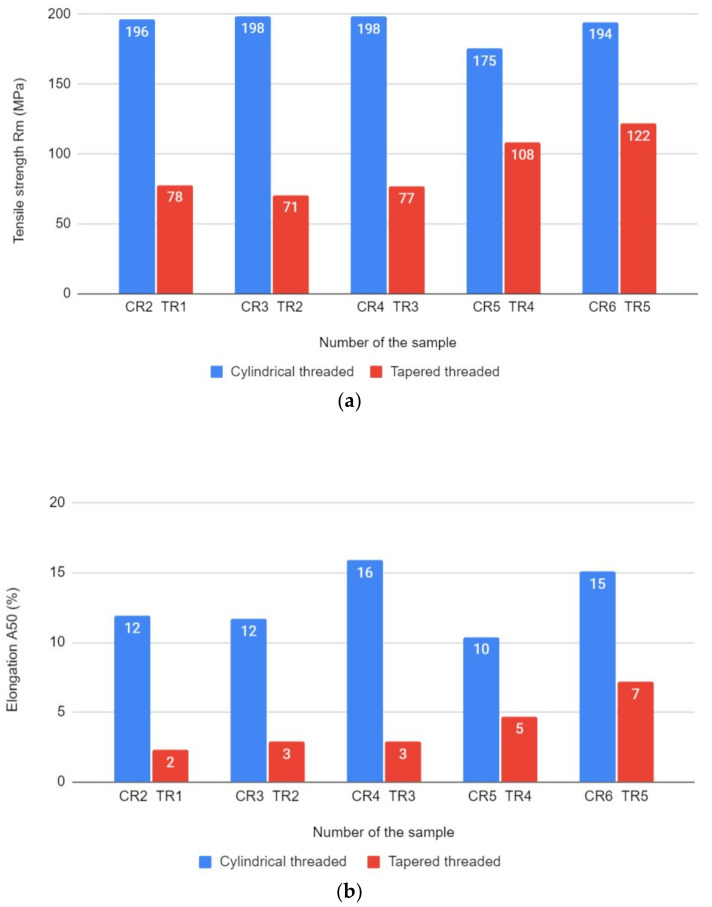
Comparison of the tool pin shape based on: (**a**) tensile strength results; (**b**) elongation results.

The results of the tensile strength tests are presented in Figure 5. Specimens were made with the same welding parameters but different pin shapes. The tensile strength results clearly point out the advantage of the mechanical properties of joints made with the cylindrical threaded pin over the properties obtained with the tapered threaded pin. The highest R_m_ result for specimens made with the cylindrical threaded pin was given for the CR3 and CR4 (both 198 MPa), but taking A_50_ into consideration, the best result was attained by the CR4 specimen (16%). However, the results for this particular pin shape are not significantly diverse, except for the CR5 specimen (R_m_ = 175 MPa and A_50_ = 10%). For the tapered threaded specimens, the best results were attained by the TR5 specimen, as both values shown–R_m_ (122 MPa) and A_50_ (7%)–were the highest. Nevertheless, the best R_m_ result given for the tapered threaded pin (TR5) was approximately 37% lower than the result attained using the same parameters but with the cylindrical threaded pin (CR6). Moreover, in a comparison of joints made with the cylindrical threaded and base metal, the percentage ratio was at least 77% (for CR5) and, in the best case, 88% (for CR3 and CR4). For the tapered threaded pin, the ratio was 31% for TR2 and 56% for the TR5. The results prove that the cylindrical threaded pin achieves significantly better results than the tapered threaded pin.

**Figure 6:**
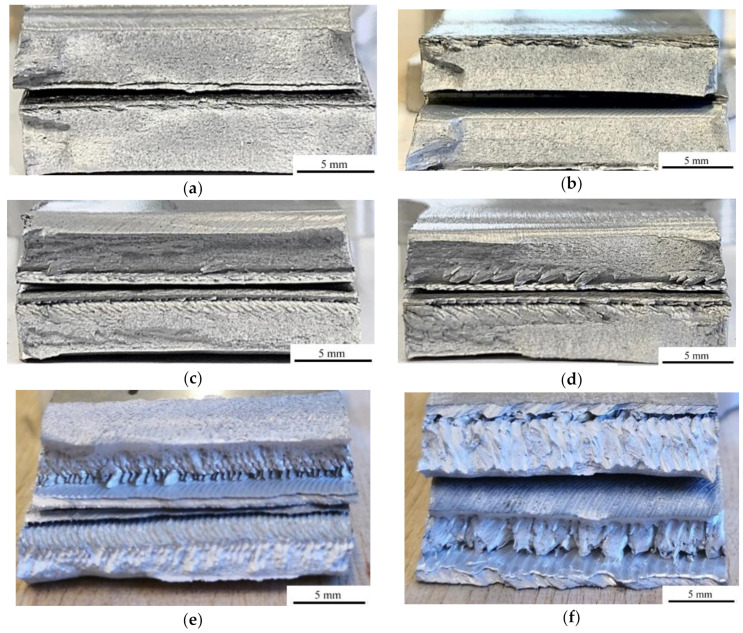
Fractographic images: (**a**) CR2; (**b**) CR3; (**c**) CR4; (**d**) CR5; (**e**) TR2; (**f**) TR5.

Selected fractographic images are presented in Figure 6. Each picture presents a cross-section of two broken pieces stacked together. For joints CR2 and CR3, full penetration was obtained (Figure 6a,b). The lack of penetration in the other joints may have been caused by the plunge depth of the tool being too low as it was proven to be one of the causes of the defect. If the plunge depth is too low, the material cannot be stirred on the complete surface of bonded metals [25]. In the joints made with the cylindrical threaded pin, a ductile fracture appeared. It suggests that the material was heated and plasticized properly (Figure 6c,d). Voids appeared in the joints made with the tapered threaded pin (Figure 6e,f). Voids are a common defect in the FSW joints [24,34,35,39,56]. This occurred even when the appearance of the joint was good [48]. The voids were present all across the width of the joint. The fracture is not ductile or plastic but confirms the poor performance of A_50_ for joints made with the tapered threaded pin (e.g., A_50_ = 3% for the TR2 specimen). The defects are the cause of the poor mechanical properties of these joints. Due to the tapered shape of the pin and insufficient heat generation, the material could not be stirred enough to obtain a consistent joint.

#### 3.2.2. Comparison of the Welding Parameters

**Figure 7:**
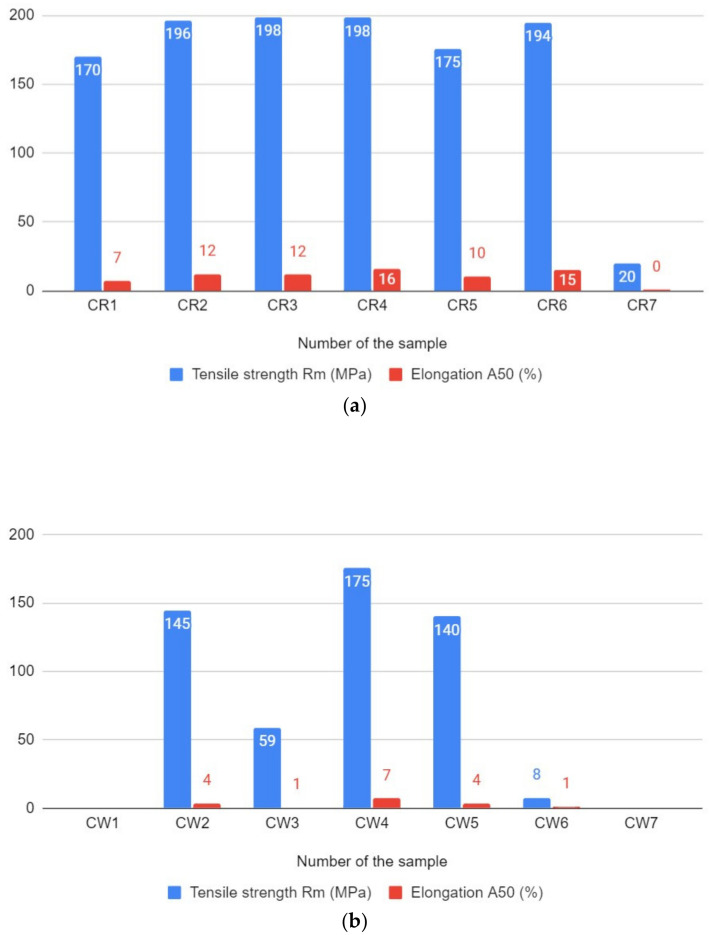
The graphs of tensile strength and elongation results depending on: (**a**) the welding speed; (**b**) the rotational speed.

The results of the tensile strength tests are presented in Figure 7. The first graph (Figure 7a) presents the R_m_ and A_50_ results for specimens made at different welding speeds but the same rotational speed (475 rpm). The R_m_ values are similar, but the CR4 and CR6 are the specimens that stand out due to their high A_50_ values, 16% and 15%, respectively. For CR6, it was 86% of the tensile strength of the base metal. The lowest mechanical properties were attained by specimen CR7, approximately R_m_ = 20 MPa and A_50_ = 0%. The percentage ratio for CR7 was 9% of the tensile strength of the base metal. It was concluded that the best mechanical properties of joints produced at a rotational speed of 475 rpm were obtained at welding speeds of 300 and 475 mm/min However, the difference between the results is not significant, so it can be stated that the range of values of the welding speed can be applied to give the relevant joint the appropriate mechanical properties.

The second graph (Figure 7b) presents Rm and A_50_ results for specimens made at different rotational speeds but the same welding speed (300 mm/min). The results are ambiguous: the figures for CW3 and CW6, in particular, are significantly lower than all the other figures. Nevertheless, the highest mechanical properties were attained at a rotational speed of 290 rpm (CW4, R_m_ = 175 MPa, A_50_ = 7%), although this figure is still 12% lower than the CR4 figure obtained using similar parameters.

**Figure 8:**
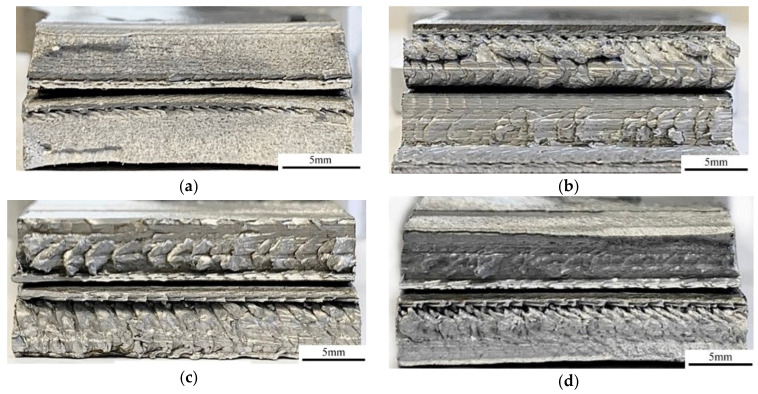
Fractographic images: (**a**) CR6; (**b**) CR7; (**c**) CW3; (**d**) CW4.

Selected fractographic images are presented in Figure 8. Each picture presents a cross section of two broken pieces stacked together. Besides CR2 and CR3, the CR6 specimen is characterized by a ductile fracture, but full penetration was not obtained in this case. As for the previous specimens, the plunge depth of the tool may be too low. In the CW4 specimen, a more brittle fracture as well as some voids appeared, which was the cause of slightly poorer mechanical properties of the joint produced. A brittle fracture was also revealed in the CR7 and CW3 specimens. The fractographic results confirm the poor mechanical properties of these joints as the welded material is not fully stirred and voids, as well as welding tool marks, are clearly visible. Especially in the case of the CR7, the brittleness might be caused by relatively low heat generation in the relevant joint, so the materials could not be stirred properly. Low heat input was studied not only in FSW but also in underwater welding as well as traditional friction welding, and in each process, it was found to cause the brittle structure of the joint produced [3,29,58,59,60]. The results reveal that rotational speed may be a more important parameter than welding speed in improving welding conditions and, in addition, joint properties.

## 4. Summary

In this paper, FSW welding conditions were examined with a view to improving the mechanical properties of EN AW-3004 joints. This material has not been joined by FSW method earlier. This entailed researching the effect of the shape of the tool pin on the joint produced was researched as well as welding parameters: welding and rotational speeds. Five joints with a tapered threaded pin and 14 with a cylindrical threaded pin were performed. Seven joints with a cylindrical threaded pin were produced at the same rotational speed and different welding speeds, and seven more joints at the same welding speed and different rotational speeds. VT and tensile strength tests were performed.

The aim of the work was achieved. Significant differences were observed in the effect of pin shape on the joints produced. Joints made with a tapered threaded pin showed poor mechanical properties, although the surface appearance was promising (mostly without excess material outflow). Fractographs of these joints revealed a brittle structure, voids and lack of penetration to be the causes of poor tensile strength and elongation. On the other hand, despite a higher amount of material outflow, joints made with a cylindrical threaded pin attained 88% of figures for the base metal without the aforementioned defects.

As for welding parameters, a range of welding speed values made it possible to obtain good mechanical properties and a ductile fracture at a rotational speed of 475 rpm. However, the mechanical properties of joints made with different values of rotational speed were not consistent at a welding speed of 300 mm/min, as defects including voids and lack of penetration were revealed. In fact, none of the specimens attained such good mechanical properties as the previous joints. This may have been caused by poor heat distribution which made it impossible to stir the material properly. With this in mind, the conclusion is that rotational speed may be the key parameter in improving the mechanical properties of FSW joints. It confirmed previous investigations that welding parameters are responsible for mechanical properties of FSW joints [61]. Moreover, performed investigations allow determination of the proper FSW welding parameters for EN AW-3004 alloy. It will allow joining of the investigated material in practical applications with good mechanical properties of the joints.

The most important conclusions to be drawn from research above are:

1. Most of the joints were produced with material outflow on the retreating side. The lowest amount was observed in the joints made with a tapered threaded tool pin.
2. Tensile strength and elongation figures were higher for joints made with a cylindrical threaded tool pin than those made with a tapered threaded pin. The latter showed voids and lack of penetration.
3. The tapered pin and insufficient heat generation were unable to produce a sound joint.
4. On the basis of the tensile strength tests, the best result was achieved at the same rotational speed and welding speed of 300 mm/min. However, the results for different joints were similar and there are a variety of welding speeds that make it possible to obtain good mechanical properties at a rotational speed of 475 rpm, ranging from about 150 to 475 mm/min.
5. At the same welding speed, the best joint was produced at a rotational speed of 290 rpm. Nevertheless, its tensile strength was lower than that of the joint made with 475 rpm and 300 mm/min.
6. Rotational speed may be the key parameter in improving welding conditions and the mechanical properties of aluminium alloy joints.

## Figures and Tables

**Table 1:** Chemical composition and mechanical properties of EN AW-3004 according to PN-EN 573-3:2019-12 [51] and EN 4852: 2016 + A1:2018 [52], wt%.

Mg (%)	Mn (%)	Fe (%)	Si (%)	Cu (%)	Zn (%)	Al (%)	Tensile Strength, R_m_ (MPa)	Elongation, A_50_ (%)
0.80–1.30	1.00–1.50	≤0.70	≤0.30	≤0.25	≤0.25	balance	≥155	≥16

**Table 2:** Welding parameters.

Symbol of the Specimen	Welding Speed (mm/min)	Rotational Speed (rpm)	The Tool Pin’ Shape
CW1	300	115	Cylindrical threaded
CW2		155	
CW3		205	
CW4		290	
CW5		360	
CW6		680	
CW7		925	
CR1	95	475	
CR2	150		
CR3	235		
CR4	300		
CR5	375		
CR6	475		
CR7	600		
TR1	150		Tapered threaded
TR2	235		
TR3	300		
TR4	375		
TR5	475		

## Data Availability

Data is contained within the article.
